# Editorial on participating in the Journal of Brachial Plexus and Peripheral Nerve Injury

**DOI:** 10.1186/1749-7221-2-8

**Published:** 2007-03-20

**Authors:** Jörg Bahm

**Affiliations:** 1 Reconstructive Microsurgery Unit, Franziskushospital, Aachen, Germany

## 

I am grateful to Rolfe Birch who gave me the opportunity to participate as a board member in a journal dedicated to brachial plexus surgery, as this field concerns my daily clinical and scientific activities over the last ten years.

Six months ago, I discovered the online *Journal of Brachial Plexus and Peripheral Nerve Injury *and was pleased by the idea of such a specific journal, providing open access and facilitating reaching colleagues all over the world, where our traditional and respected journals might not go. I also appreciate the technical opportunity, feasible within this journal, of publishing video documentation, as our operative results might be actually better expressed through this medium.

I have to congratulate Dr Nath for his excellent initiative. I propose my input for this journal:

- I expect that the journal will fulfill criteria of a scientifically honest, balanced and innovative platform for all interested colleagues;

- I believe that an online journal with free access in all countries has large possibilities to share data, high quality photos and videos with critical readers

- My goal is to join the Journal and uphold international scientific standards and basic rules when sharing high level medical and scientific expertise.

I would like to encourage all colleagues to participate in the discussion by sharing their experience, comments, criticisms, starting with questions, observations, empiric conclusions and scientific contributions.

The *JBP&PNI *started as an initiative to be an open-minded platform for an exchange of ideas and techniques in this particular field of clinics and surgery, brachial plexus pathology and related issues in severe peripheral nerve injuries.

Colleagues from all countries who are involved in these treatment plans should feel free to communicate their experience and analysis of the existing literature. Senior colleagues should share their comments about trials and pitfalls, for the benefit of our continued education and thus better serve patients hit by severe nerve injuries, either children or adults.

During the past 10 years, I have become aware of how many controversies might exist even between international colleagues sharing one particular medical problem, and this is for many reasons. I believe, however, that we should overcome these differences just by focusing on common, noble targets of scientific education, giving our very best, and respecting established rules that good medical practice has taught us.

Jörg Bahm MD

February 2007

